# Supplementary material for: Identification of an adhesive interface for the non-clustered δ1 protocadherin-1 involved in respiratory diseases
Source: Commun Biol. 2019 Sep 30;2:354. doi: 10.1038/s42003-019-0586-0 (PMC6769022; doi:10.1038/s42003-019-0586-0)
Supplement: Supplementary file 1 — Supplementary Information [file 42003_2019_586_MOESM1_ESM.pdf]

Supplementary Figures

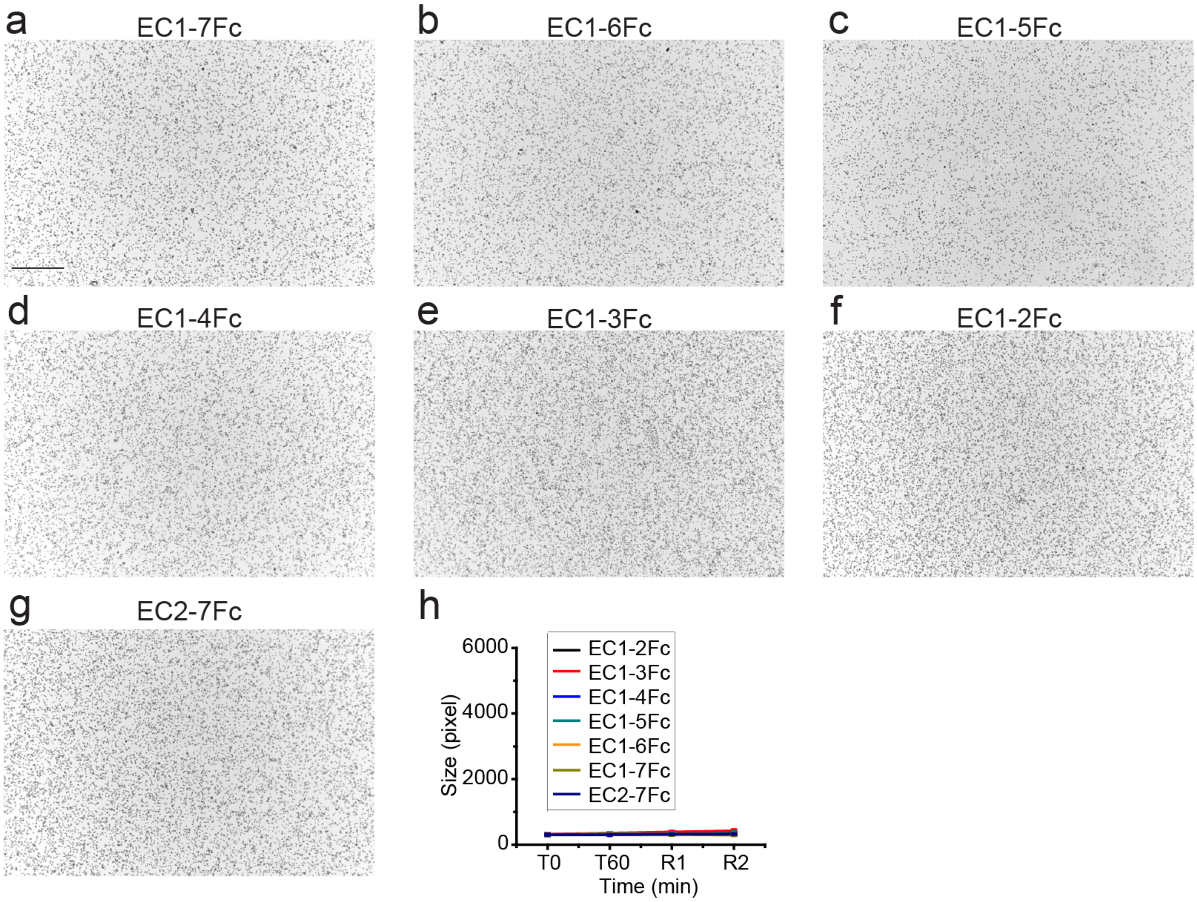

Supplementary Figure 1. PCDH1 does not mediate adhesion in the absence of calcium (2 mM EDTA). a-g Protein G beads coated with full length (**a**) and truncated versions (**b-g**) of the PCDH1 extracellular domain imaged after incubation for 1 hr followed by rocking for 2 min in the absence of calcium. Bar – 500 µm. **h** Mean aggregate size for full length and truncated fragments of PCDH1 at T0 (t = 0 min), after 1 hr of incubation, T60 (t = 60 min) followed by rocking for 1 min (R1) and 2 min (R2). Error bars are standard error of the mean (n = 4 independent experiments for all constructs except for PCDH1 EC1-7Fc with n = 3 independent experiments).

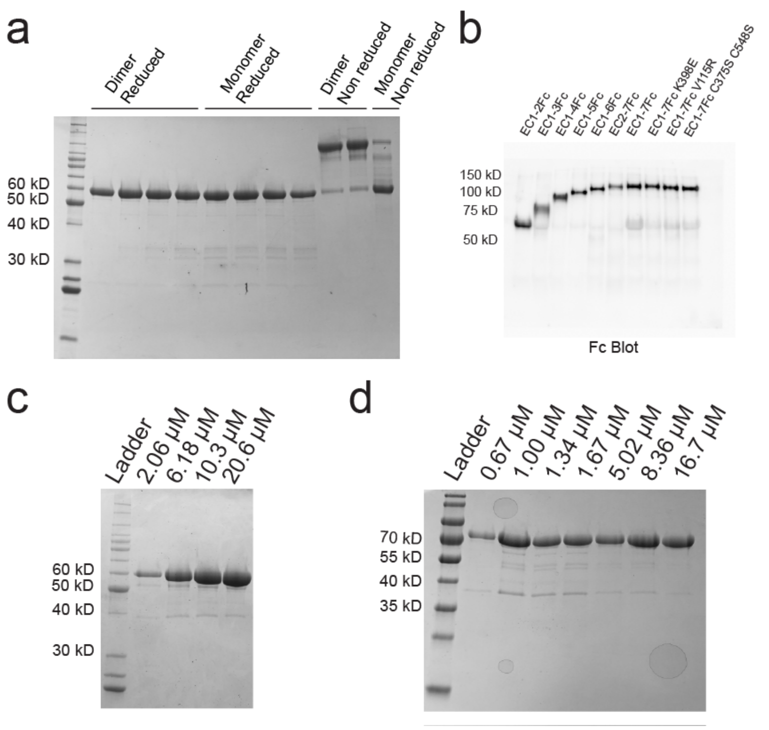

Supplementary Figure 2. Analyses of protein disulfide-bond formation, purity, and integrity. a SDS-PAGE analyses of SEC fractions for PCDH1 EC1-4 showing a cysteine-dependent dimer of wild-type PCDH1 EC1-4. **b** Western blot shows efficient expression of truncated, full length, and full-length mutant PCDH1 protein fragments. Lanes 1-7 were used in Fig. 1, lanes 7 and 10 were used in Fig. 2, and lanes 7 to 9 were used in Fig. 5. **c-d** SDS-PAGE analyses of AUC samples show that PCDH1 EC1-4 C375S (**c**) and PCDH1 EC1-5 C375S C548S (**d**) were pure and did not degrade after AUC. Lanes 1 and 6 to 8 (omitted) are shown in Supplementary Fig. 6g. Samples were not loaded in equal amounts for running the gel.

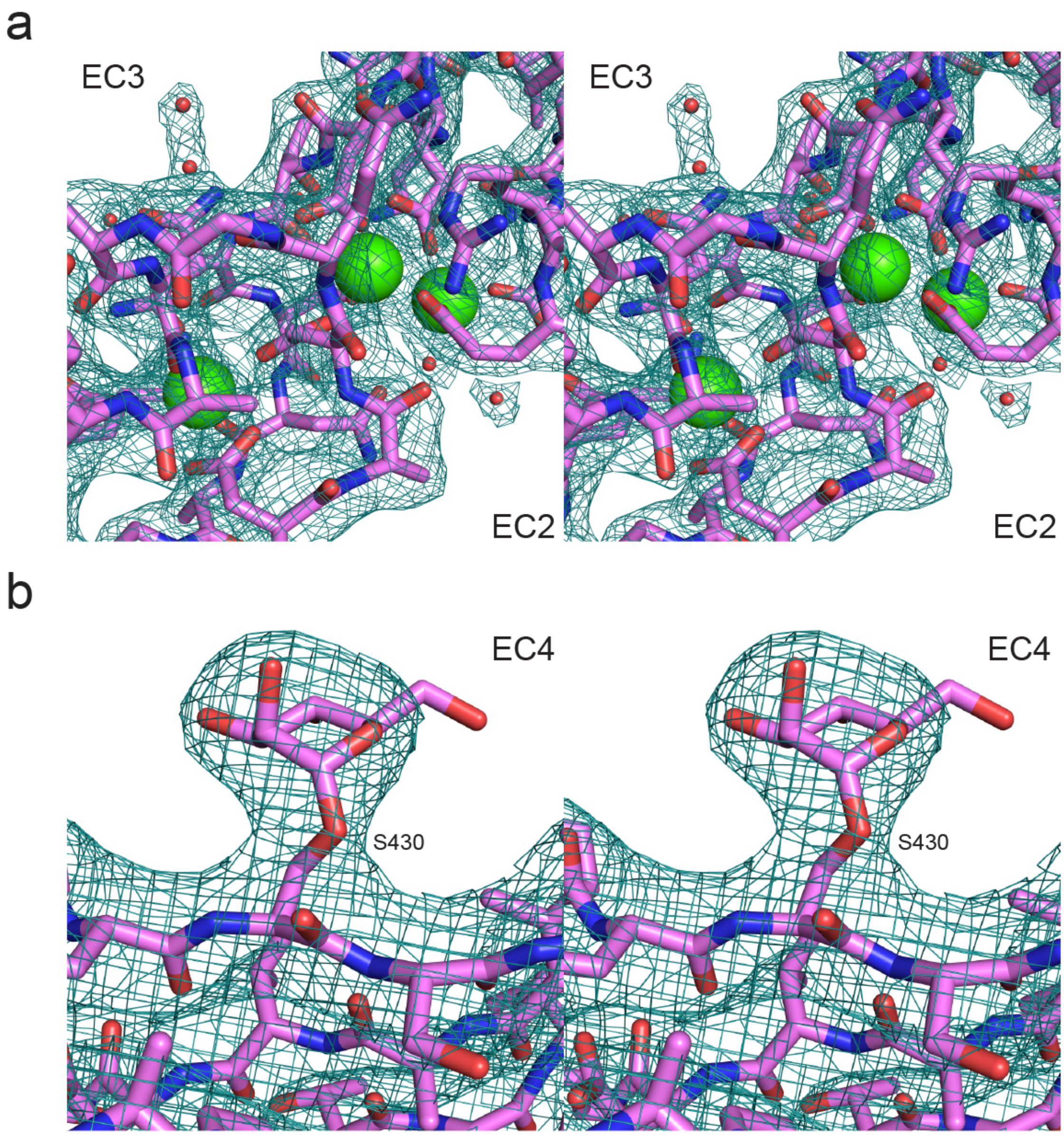

Supplementary Figure 3. Electron density maps of PCDH1 structures. **a** Stereo view of the $2F_O$-Fc electron density map (teal mesh) of the EC2-3 linker region in the PCDH1 EC1-4bc structure (2.85 Å; 6BX7) contoured at 2.0 σ. **b** Stereo view of the $2F_O$-Fc electron density map (teal mesh) of the mannose sugar molecule in the PCDH1 EC1-4mc structure (3.15 Å; 6MGA) contoured at 1.3 σ.

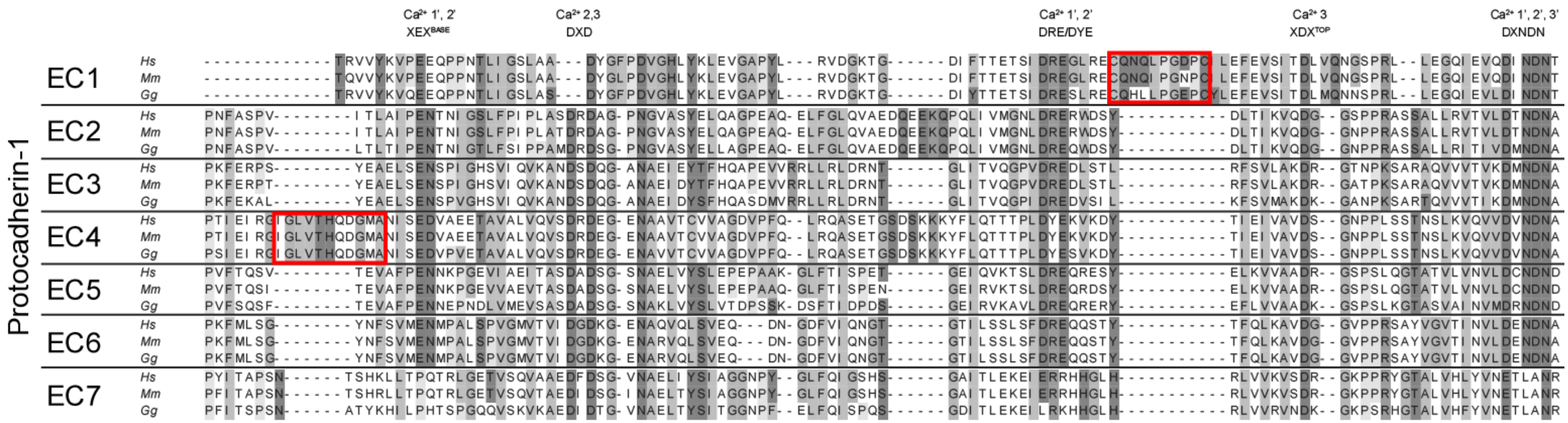

Supplementary Figure 4. Sequence alignment of human (*hs*), mouse (*mm*), and chicken (*gg*) PCDH1 EC repeats. All 7 EC repeats for each species are aligned to each other (EC1 to EC7). Conserved calcium-binding motifs are labeled. Red boxes show the cysteine loop in EC1 and the δ insertion in EC4.

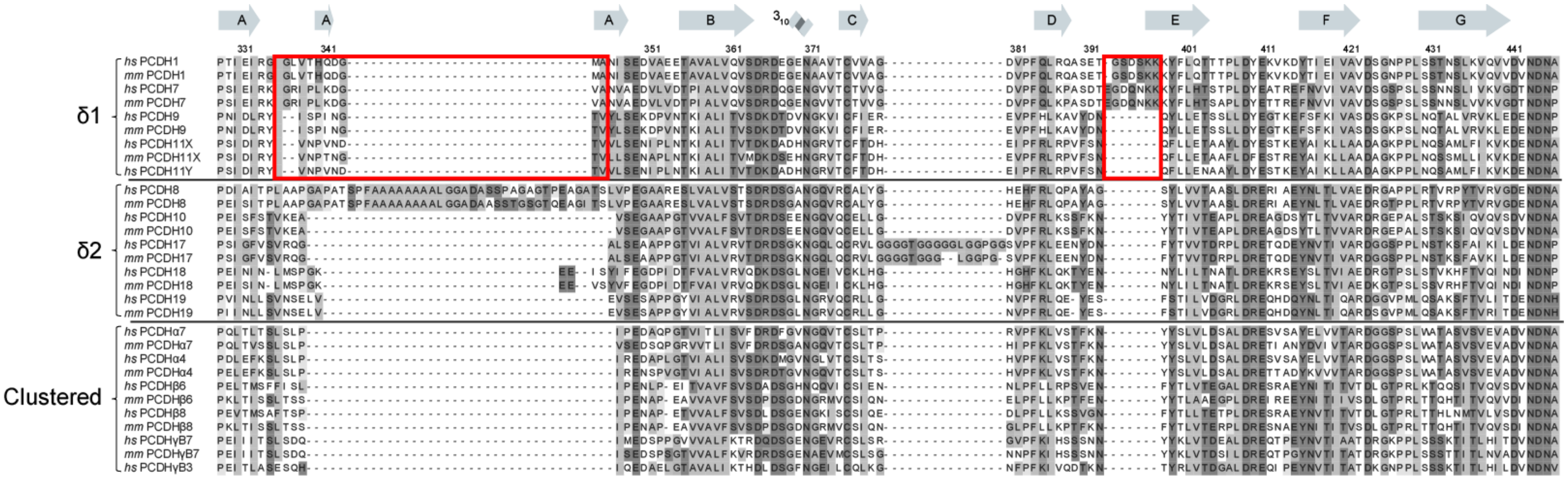

Supplementary Figure 5. Sequence alignment of repeat EC4 from selected δ and clustered protocadherins. Secondary structure from *hs* PCDH1 EC4 is indicated on top of the alignment. Red boxes show the δ insertion and a unique DE loop in PCDH1. The accession numbers for the protein sequences used are given in Supplementary Tables 2 and 3.

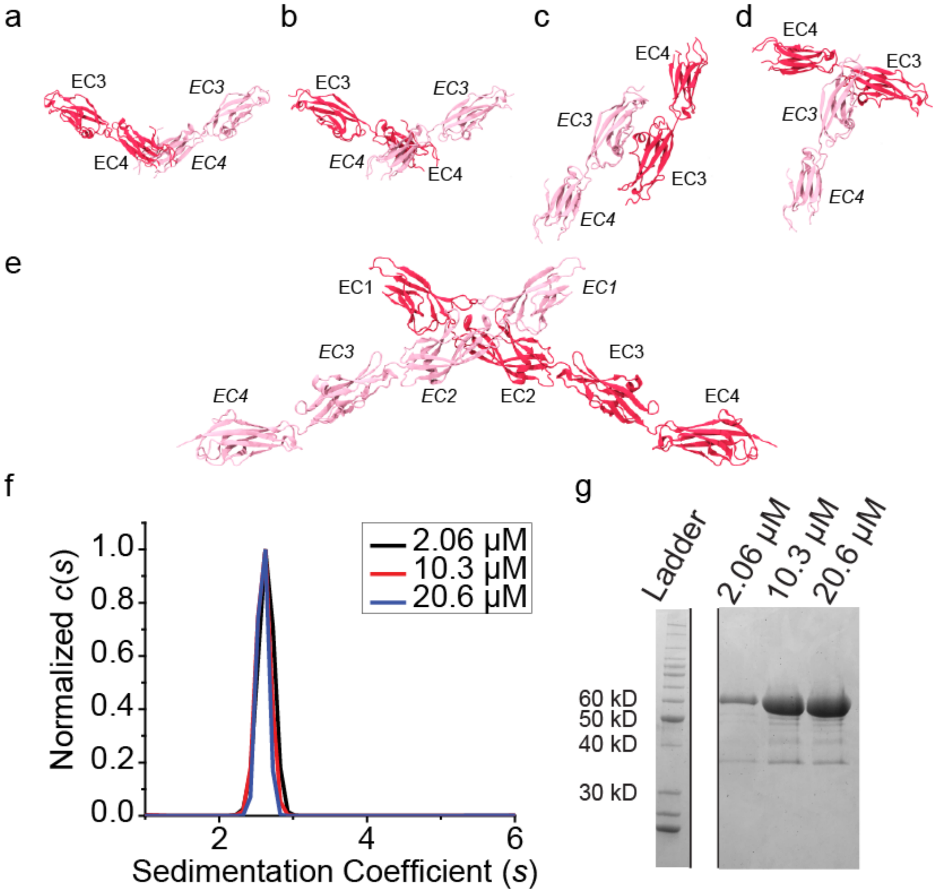

Supplementary Figure 6. Crystal contact analyses and oligomerization state of a PCDH1 mutant. a-d Crystal contacts in the PCDH1 EC3-4bc structure. Interface areas are ~517 Å^2 (**a**), ~473 Å^2 (**b**), ~362 Å^2 (**c**), and 307 Å^2 (**d**), all bellow an empirical cut-off of 856 Å^2 used to distinguish biological interfaces from crystal contacts. Crystal contact shown in (**a**) is incompatible with the *trans* PCDH1 I1 interface, while the one shown in (**b**) is incompatible with the *trans* PCDH1 I2 interface. Crystal contact shown in (**c**) is distinct from the EC3:EC3 contact in the PCDH1 I2 interface. **e** Crystal contact in PCDH1 EC1-4 structures involving EC2 repeats. Interface area is ~486 Å^2. Adhesion mediated by PCDH1 requires EC1, suggesting that these small interfaces are non-physiological or transient. **f** AUC of PCDH1 EC1-4 C375S K398E (n = 2 independent experiments) shows only a monomeric peak for a range of concentrations between 2.06 μM and 20.6 μM. Normalized $c(s)$ has been plotted against sedimentation coefficient (s). The bacterially produced PCDH1 EC1-4 C375S K398E protein fragment does not dimerize in solution. **g** SDS-PAGE analysis shows that samples for PCDH1 EC1-4 C375S K398E were pure and did not degrade after AUC. Split from gel shown in Supplementary Fig. 2c. Samples were not loaded in equal amount for the SDS-PAGE.

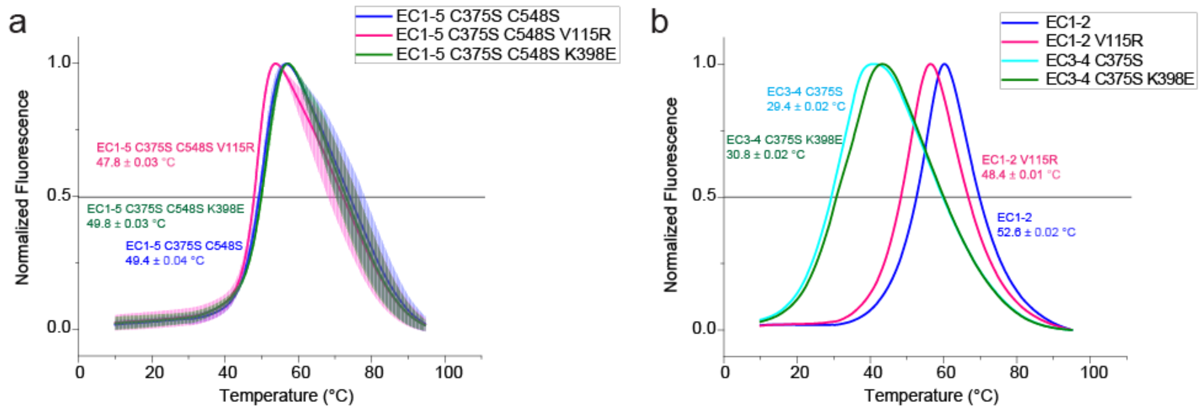

Supplementary Figure 7. Melting temperatures of PCDH1 mutants determined using differential scanning fluorimetry show that their thermal stability is comparable. a Melting temperature of PCDH1 EC1-5 C375S C548S (49.4 ± 0.04 °C), EC1-5 C375S C548S V115R (47.8 ± 0.03 °C), and EC1-5 C375S C548S K398E (49.8 ± 0.03 °C) are comparable. Curves are average signal for each protein fragment with vertical bars representing standard deviation from the mean (*n* = 3 independent experiments). **b** Melting temperatures of PCDH1 EC1-2 V115R (48.4 ± 0.01 °C) is slightly smaller than WT (52.6 ± 0.02 °C). Melting temperatures of PCDH1 EC3-4 C375S (29.4 ± 0.02 °C) and EC3-4 C375S K398E (30.8 ± 0.02 °C) are comparable. Curves are average signal for each protein fragment (*n* = 2 independent experiments).

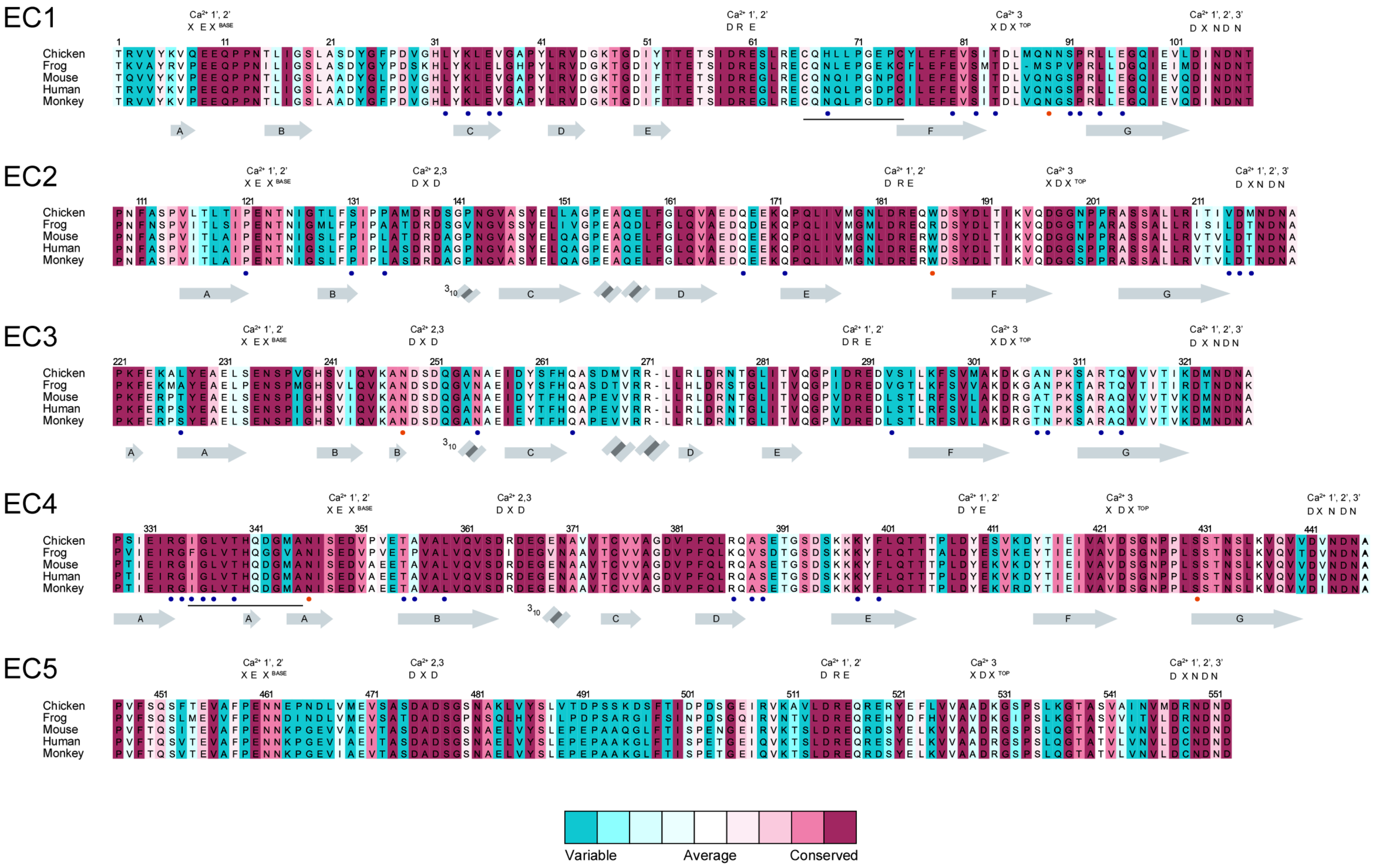

Supplementary Figure 8. Sequence alignment of PCDH1 EC1-5. Alignment of chicken, frog, mouse, human, and monkey sequences for PCDH1 EC1-5. Residues are colored according to conservation based on ConSurf and an alignment of sequences from 87 species. Conserved calcium-binding motifs are shown on top of the alignment and labeled. Interfacing residues are marked as blue dots. Predicted and observed glycosylation sites are marked as orange dots. The cysteine disulfide loop in EC1 and the δ insertion in EC4 are underlined. The accession numbers for the protein sequences of each species used are given in Supplementary Table 3.

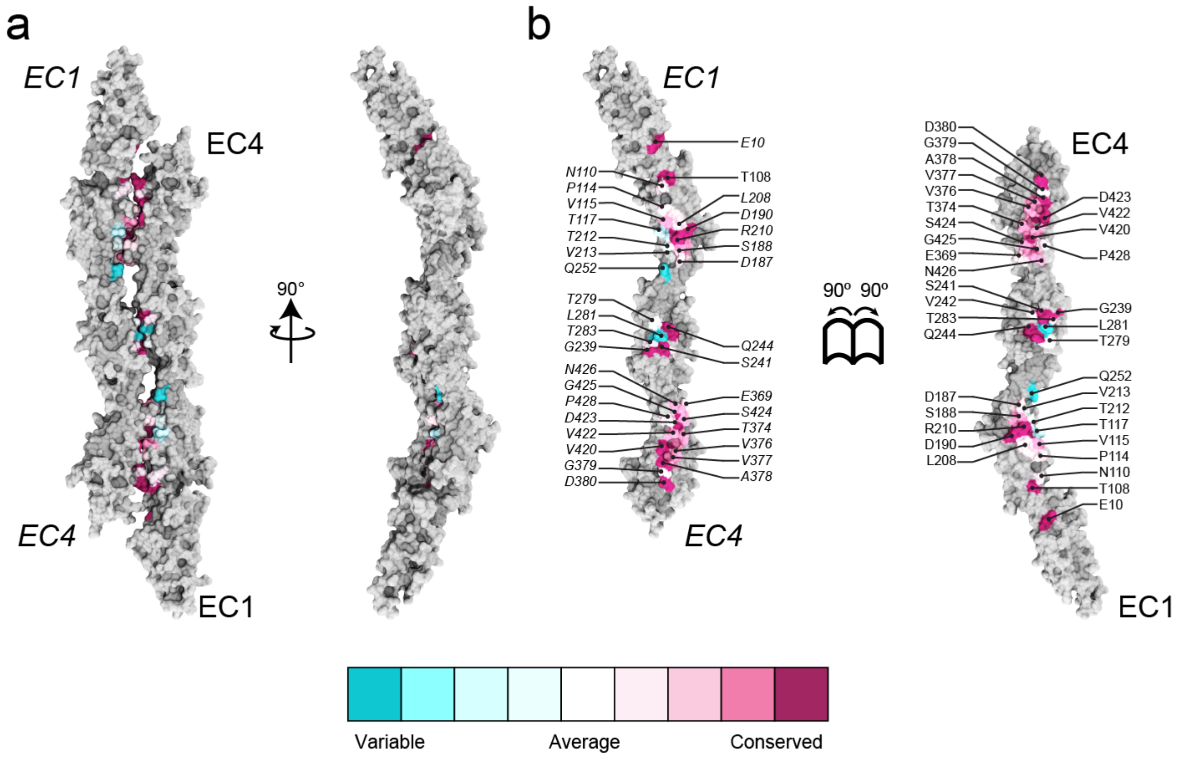

Supplementary Figure 9. Conservation of residues at the crystallographic PCDH1-I2 interface. a Molecular surface representation of the PCDH1-I2 antiparallel dimer. Two perpendicular views are shown. **b** Interaction surface exposed with interfacing residues listed and colored according to sequence conservation among 87 species. The accession numbers for the protein sequences of each species used are given in Supplementary Table 3.

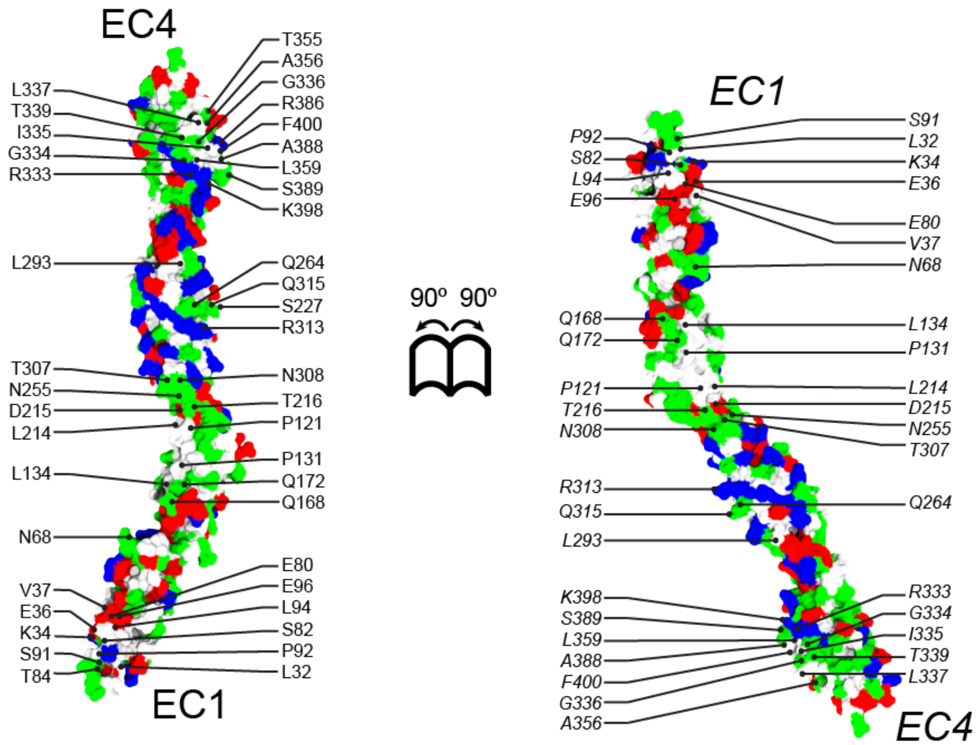

Supplementary Figure 10. **PCDH1 antiparallel EC1-4 dimer interface involves charged, hydrophilic, and hydrophobic residues.** Molecular surface representation of the PCDH1 I1 antiparallel dimer with interfacial residues exposed and labeled. Surface is colored according to residue type (apolar: white; polar: green; negatively charged: red; positively charged and histidines: blue).

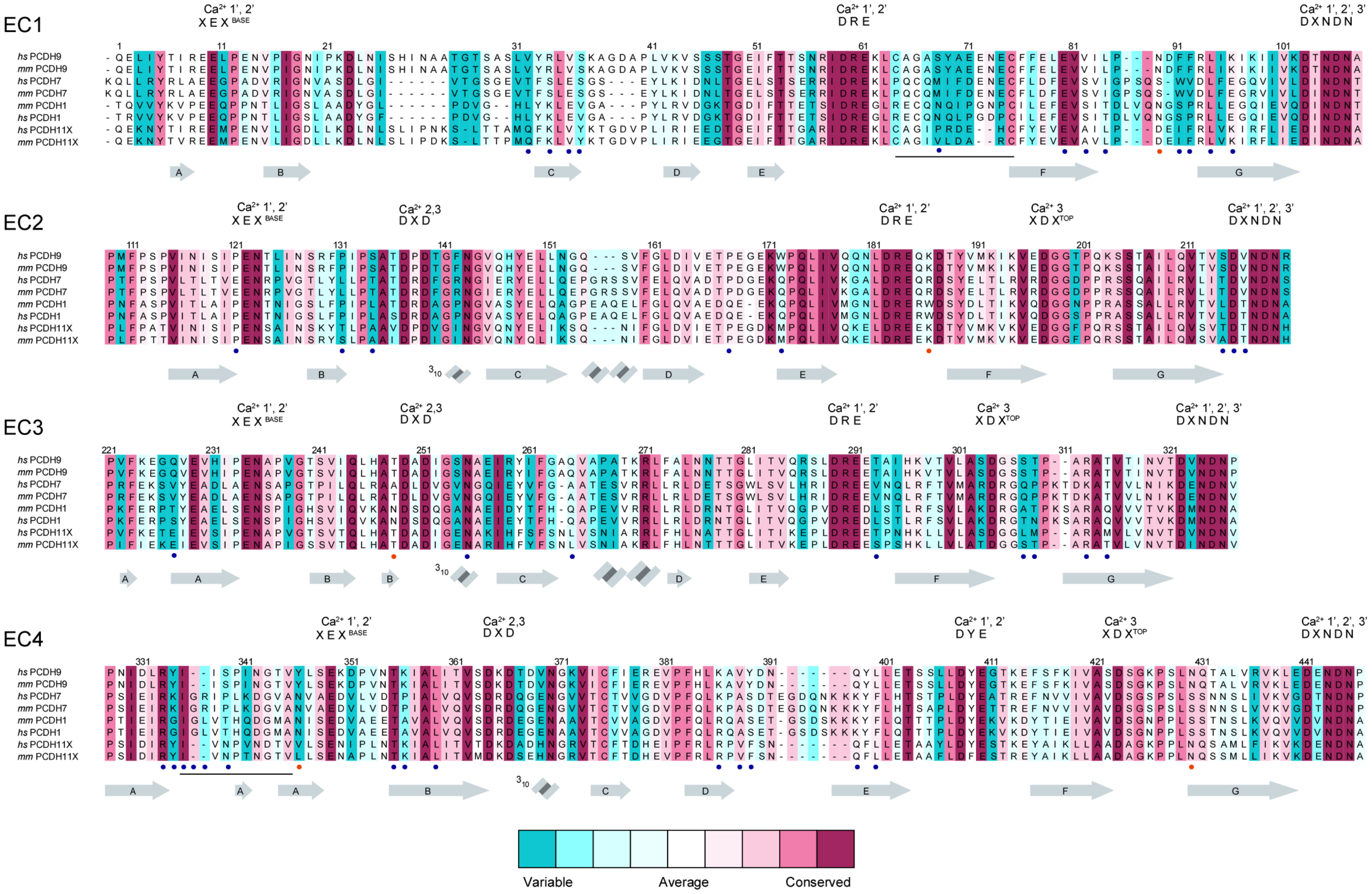

Supplementary Figure 11. Sequence alignment of *hs* and *mm* PCDH9, PCDH7, PCDH1, and PCDH11X EC1 to EC4. Calcium-binding motifs are labeled. Interfacing residues are marked as blue dots. Predicted and observed glycosylation sites are marked as orange dots. The cysteine disulfide loop in EC1 and the δ insertion in EC4 are underlined. The accession numbers for the protein sequences of each species used are given in Supplementary Table 4.

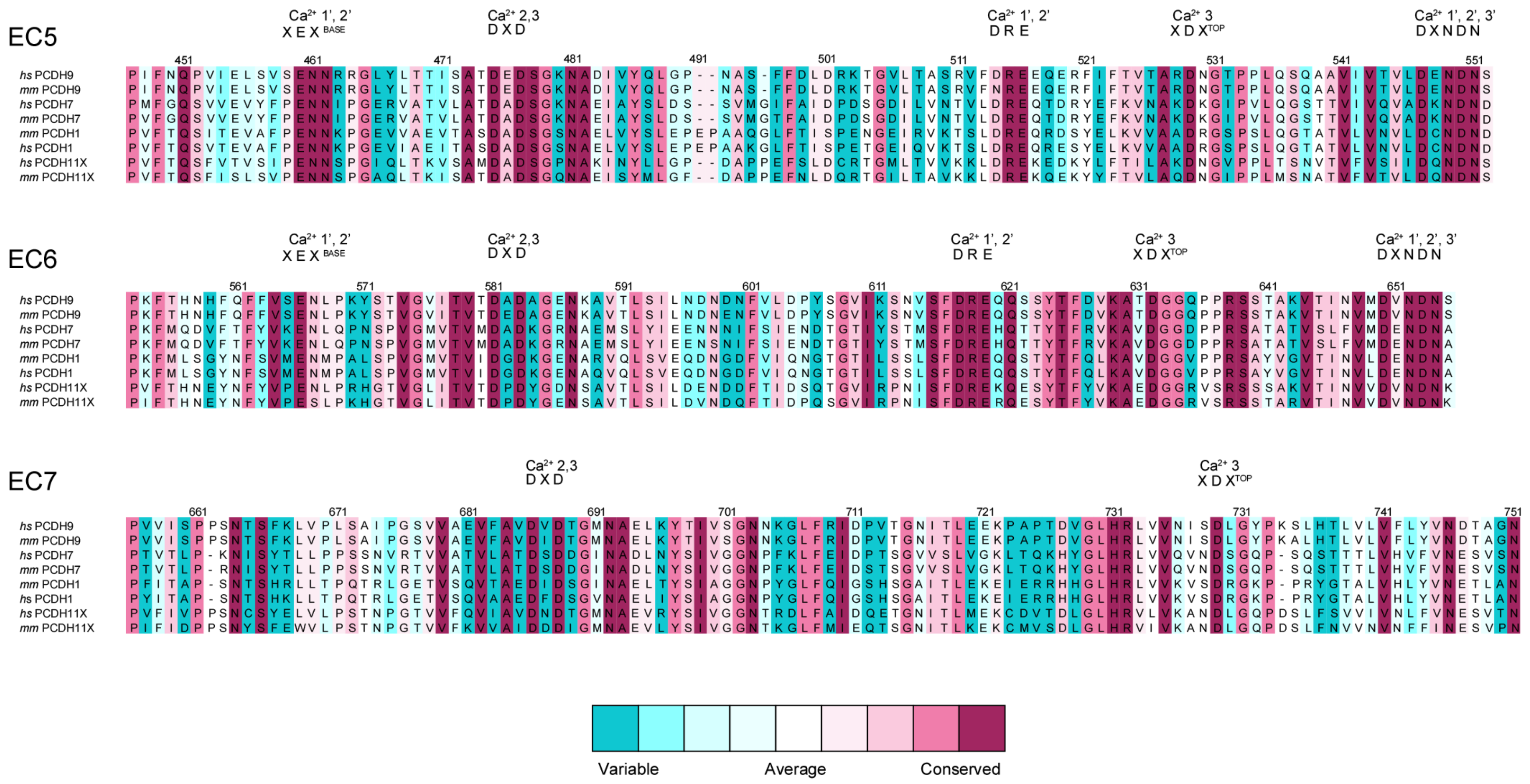

Supplementary Figure 12. Sequence alignment of *hs* and *mm* PCDH9, PCDH7, PCDH1, and PCDH11X EC5 to EC7. Calcium-binding motifs are labeled. The accession numbers for the protein sequences of each species used are given in Supplementary Table 4.

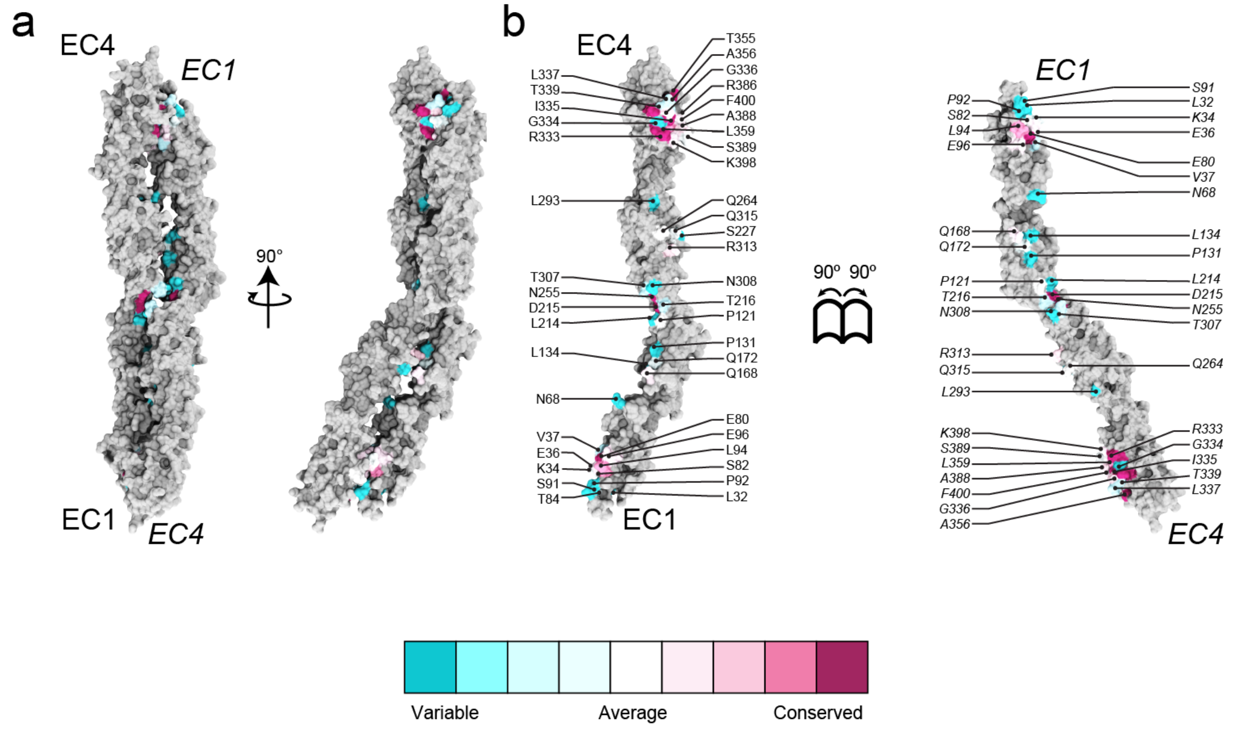

Supplementary Figure 13. Conservation of residues among all δ1 protocadherins at the crystallographic PCDH1-I1 interface. **a** Molecular surface representation of the PCDH1-I1 antiparallel dimer. Two perpendicular views are shown. **b** Interaction surface exposed with interfacing residues listed and colored according to sequence conservation across different δ1 protocadherins. The accession numbers for the protein sequences of each species used are given in Supplementary Table 4.

δ1 Protocadherins Identity

***hs*PCDH1**	100%			
***hs*PCDH7**	53.54%	100%		
***hs*PCDH9**	45.27%	43.92%	100%	
***hs*PCDH11X**	42.91%	38.13%	58.98%	100%
	***hs*PCDH1**	***hs*PCDH7**	***hs*PCDH9**	***hs*PCDH11X**

δ1 Protocadherins Similarity

***hs*PCDH1**	100%			
***hs*PCDH7**	65.87%	100%		
***hs*PCDH9**	55.90%	56.70%	100%	
***hs*PCDH11X**	53.67%	50.17%	72.91%	100%
	***hs*PCDH1**	***hs*PCDH7**	***hs*PCDH9**	***hs*PCDH11X**

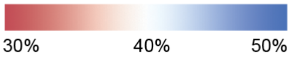

Supplementary Figure 14. δ1 protocadherins are similar in sequence. Identity and similarity matrices of δ1 protocadherins were calculated with the SIAS server using their EC1-7 extracellular domains.

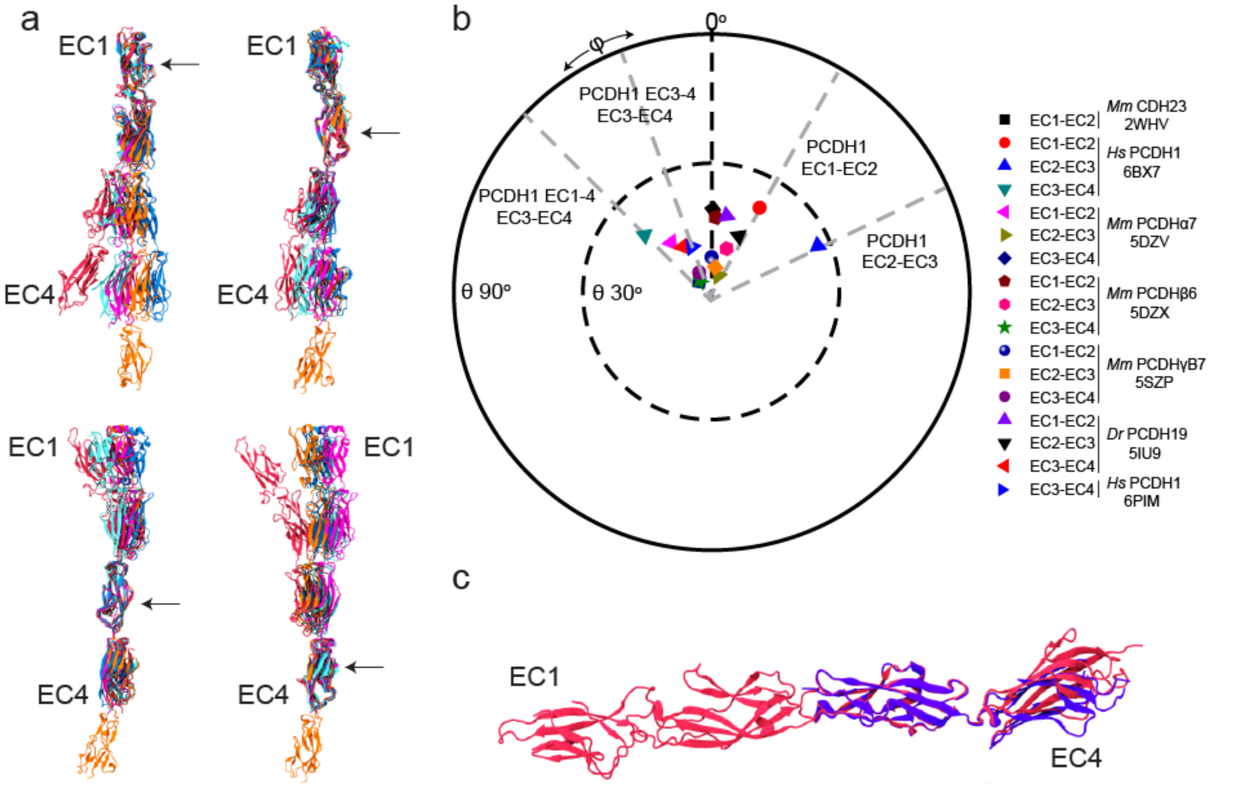

Supplementary Figure 15. **PCDH1 (δ1) is more curved than the clustered (α, β, γ) and PCDH19 (δ2) protocadherins. a** Alignments of ECs of PCDH1 (red), PCDHα7 (orange), PCDHβ6 (magenta), PCDHγB7 (blue) and PCDH19 (cyan) show that PCDH1 is more twisted than the others. The top left and right panels have EC1s and EC2s aligned, respectively. The bottom left and right panels have EC3s and EC4s aligned, respectively. Arrows indicate aligned EC. **b** Orientation of tandem EC repeats for listed cadherins shows that PCDH1 angles are different. The N-terminal EC repeat for labeled structures was used as reference and aligned to the *z*-axis. CDH23 EC1-2 was used to define φ = 0°. The azimuthal angle (φ) observed for PCDH1 EC2-3 is unique. The azimuthal angles for EC3-EC4 in the PCDH1 structures PCDH1 EC1-4bc and PCDH1EC3-4bc are similar, but not identical, and the tilt is more pronounced in the PCDH1 EC1-4bc structure. This change in conformation reflects some flexibility that might be relevant during complex formation. **c** Alignment of repeats EC3 in the PCDH1 EC1-4bc (red) and PCDH1 EC3-4bc (violet) structures shows that the EC3-EC4 tilt is less pronounced in the PCDH1 EC3-4bc structure.

Supplementary Tables

Supplementary Table 1. Comparison of linker regions length and sequences among classical cadherins and δ1, δ2, and clustered protocadherins.

	PDB Code	Ca^{2+} 2-3 (Å)[1]	Ca^{2+} 1-3 (Å)[2]	$D_{C\alpha} – X_N$ (Å)[3]	Sequence Alignment
Mm CDH1 EC1-2	3Q2V classical	6.70	9.34	12.33	VTDQNDNRPEFTQ
Mm CDH1 EC2-3		7.17	10.79	12.11	VKDINDNAPVFNP
Mm CDH1 EC3-4		7.37	10.01	**13.64**	VVDVNEA-PIFMP
Mm CDH1 EC4-5		7.17	8.42	12.30	LLDVNDNAPIPEP
Mm CDH2 EC1-2	3Q2W classical	6.64	9.52	12.44	VIDMNDNRPEFLH
Mm CDH2 EC2-3		6.67	9.40	12.48	VTDVNDNPPEFTA
Mm CDH2 EC3-4		6.85	9.50	**14.42**	VIDVNEN-PYFAP
Mm CDH2 EC4-5		6.80	9.26	12.39	LLDINDNAPQVLP
Hs PCDH1 EC1-2	6BX7 δ1	6.85	9.60	12.35	VQDINDNTPNFAS
Hs PCDH1 EC2-3		6.70	9.47	12.29	VLDTNDNAPKFER
Hs PCDH1 EC3-4		6.66	9.10	12.29	VKDMNDNAPTIEI
Dr PCDH19 EC1-2	5IU9 δ2	6.61	9.42	11.72	IIDVNDNAPRFPT
Dr PCDH19 EC2-3		7.00	9.93	12.17	VIDSNDNNPVFDE
Dr PCDH19 EC3-4		6.76	8.83	12.22	VIDINDNAPEIKL
Mm PCDHα7 EC1-2	5DZV clustered	7.12	10.39	12.07	VKDINDNPPMFPA
Mm PCDHα7 EC2-3		7.13	9.30	12.11	VLDVNDNAPVFDR
Mm PCDHα7 EC3-4		7.49	8.65	12.17	VVDANDNAPQLTV
Mm PCDHβ6 EC1-2	5DZX clustered	6.83	9.71	12.73	LTDINDHSPEFPD
Mm PCDHβ6 EC2-3		7.27	9.41	12.46	VVDINDNAPEFVQ
Mm PCDHβ6 EC3-4		6.84	9.30	12.31	VLDVNDNAPKLTI
Mm PCDHγB7 EC1-2	5SZP clustered	6.75	9.90	12.05	IEDVNDHAPQFPK
Mm PCDHγB7 EC2-3		7.07	9.20	12.21	VVDANDNRPVFSQ
Mm PCDHγB7 EC3-4		6.18	8.41	12.38	VLDENDNRPEIII
Average		6.90	9.43	12.42	

[1] Distances between Ca^{2+} ions at sites 2 and 3 (Ca^{2+} 2-3).
[2] Distances between Ca^{2+} ions at sites 1 and 3 (Ca^{2+} 1-3).
[3] Distances between the Cα atoms of the first aspartate residue in the DXNDNX motif ($D_{C\alpha}$), and the backbone N atom of the last residue in the same motif (X_N) ($D_{C\alpha} – X_N$).

Supplementary Table 2. Accession numbers of δ1, δ2, and clustered protocadherin protein sequences used for studying unique features in EC4 of PCDH1.

S/N	Name	Accession Number
1	*hs* PCDH11Y	NP_116754.1
2	*hs* PCDH8	NP_002581.2
3	*mm* PCDH8	NP_067518.2
4	*hs* PCDH10	NP_116586.1
5	*mm* PCDH10	NP_001091640.1
6	*hs* PCDH17	NP_001035519.1
7	*mm* PCDH17	NP_001013775.2
8	*hs* PCDH18	NP_061908.1
9	*mm* PCDH18	NP_569715.3
10	*hs* PCDH19	NP_001171809.1
11	*mm* PCDH19	NP_001098715.1
12	*hs* PCDHα7	NP_061733.1
13	*mm* PCDHα7	NP_034087.1
14	*hs* PCDHα4	NP_061730.1
15	*mm* PCDHα4	NP_031792.1
16	*hs* PCDHβ6	NP_061762.2
17	*mm* PCDHβ6	NP_444361.1
18	*hs* PCDHβ8	NP_061993.3
19	*mm* PCDHβ8	NP_444363.1
20	*hs* PCDHγB7	NP_061750.1
21	*mm* PCDHγB7	NP_291057.1
22	*hs* PCDHγB3	NP_061747.2

Supplementary Table 3. Accession numbers of the PCDH1 EC1-7 sequences of 87 species used for studying conservation of residues.

S/N	Common name	Scientific name	Accession number
1.	Human	*Homo sapiens*	NP_115796.2
2.	Mouse	*Mus musculus*	NP_083633.2
3.	Chicken	*Gallus gallus*	XP_015149085.1
4.	Rat	*Rattus norvegicus*	XP_006222627.1
5.	Cattle	*Bos taurus*	NP_001077124.1
6.	Orangutan	*Pongo abelii*	XP_024103364.1
7.	Northern white-cheeked gibbon	*Nomascus leucogenys*	XP_012360025.1
8.	Frog	*Xenopus tropicalis*	NP_001096175.1
9.	Dog	*Canis lupus familiaris*	XP_013963480.1
10.	Chimpanzee	*Pan troglodytes*	XP_009448113.1
11.	Horse	*Eqqus caballus*	XP_023473277.1
12.	Domestic ferret	*Mustela putoriusfuro*	XP_004744802.1
13.	Brandt's bat	*Myotis brandtii*	XP_014384591.1
14.	Golden hamster	*Mesocricetus auratus*	XP_012968155.1
15.	Pig	*Sus crofa*	XP_020940599.1
16.	Rhesus monkey	*Macaca mulatta*	XP_014996578.1
17.	Upper Galilee mountains blind mole rat	*Nannispalax galili*	XP_008833733.1
18.	Beluga whale	*Delphinapterus leucas*	XP_022445669.1
19.	Hawaiian monk seal	*Neomonachus schauinslandi*	XP_021557659.1
20.	Mongolian gerbil	*Meriones unguiculatus*	XP_021506942.1
21.	Helmeted guineafowl	*Numida meleagris*	XP_021266215.1
22.	Shrew mouse	*Mus pahari*	XP_021069920.1
23.	Koala	*Phascolarctos cinereus*	XP_020860723.1
24.	Great blue-spotted mudskipper	*Boleophthalmus pectinirostris*	XP_020785977.1
25.	Central bearded dragon	*Pogona vitticeps*	XP_020654882.1
26.	Whale shark	*Rhincodon typus*	XP_020375084.1
27.	Zebu cattle	*Bos indicus*	XP_019820689.1
28.	Chinese rufous horseshoe bat	*Rhinolophussinicus*	XP_019594645.1
29.	Great roundleaf bat	*Hipposiderosarmiger*	XP_019518573.1
30.	Australian saltwater crocodile	*Crocodylus porosus*	XP_019390369.1
31.	Gharial	*Gavialis gangeticus*	XP_019362111.1
32.	Leopard	*Panthera pardus*	XP_019280068.1
33.	Asian bonytongue	*Scleropages formosus*	XP_018592904.1
34.	Blue-crowned manakin	*Lepidothrix coronata*	XP_017685910.1
35.	White-faced capuchin	*Cebus capucinus imitator*	XP_017387745.1
36.	Natal long-fingered bat	*Miniopterus natalensis*	XP_016061056.1
37.	Egyptian rousette	*Rousettus aegyptiacus*	XP_015979646.1
38.	Japanese quail	*Coturnix japonica*	XP_015731461.1
39.	Brown-spotted pit viper	*Protobothrops mucrosquamatus*	XP_015672907.1
40.	Great tits	*Parus major*	XP_015496986.1
41.	Amur tiger	*Panthera tigris altaica*	XP_015391107.1
42.	Alpine marmot	*Marmota marmota marmota*	XP_015354329.1
43.	Gecko	*Gekko japonicus*	XP_015269557.1
44.	Cheetah	*Acinonyx jubatus*	XP_014918959.1
45.	Ruff	*Calidris pugnax*	XP_014811282.1

46.	Common starling	*Sturnus vulgaris*	XP_014745102.1
47.	Donkey	*Equus asinus*	XP_014705742.1
48.	Garter snake	*Thamnophis sirtalis*	XP_013908725.1
49.	North island brown kiwi	*Apteryxaustralis mantelli*	XP_013802378.1
50.	Domestic goose	*Anser cygnoides domesticus*	XP_013033983.1
51.	Ord’s kangaroo rat	*Dipodomys ordii*	XP_012875722.1
52.	Grey mouse lemur	*Microcebus murinus*	XP_012604524.1
53.	Coquerel’s sifaka	*Propithecus coquereli*	XP_012501284.1
54.	Ma’s night monkey	*Aotus nancymaae*	XP_012317787.1
55.	Sooty mangabey	*Cerocebus atys*	XP_011945991.1
56.	Pig-tailed macaque	*Macacanemestrina*	XP_011714527.1
57.	Golden eagle	*Aquila chrysaetoscanadensis*	XP_011569959.1
58.	Large flying fox	*Pteropus vampyrus*	XP_023390699.1
59.	Arabian camel	*Camelus dromedarius*	XP_010980451.1
60.	Bactrian camel	*Camelus bactrianus*	XP_010948845.1
61.	Bison	*Bison bison bison*	XP_010848704.1
62.	Damara mole-rat	*Fukomys damarensis*	XP_010612182.1
63.	Bald eagle	*Haliaeetus leucocephalus*	XP_010577476.1
64.	Hooded crow	*Corvus cornix cornix*	XP_010408469.1
65.	Golden snub-nosed monkey	*Rhinopithecus roxellana*	XP_010386558.1
66.	East African grey-crowned crane	*Balearicaregulorum gibbericeps*	XP_010299558.1
67.	White-throated tinamou	*Tinamusguttatus*	XP_010224802.1
68.	Chuck-will’s-widow	*Antrostomus carolinensis*	XP_010162738.1
69.	Macqueen’s bustard	*Chlamydotis macqueenii*	XP_010124265.1
70.	Chimney swift	*Chaetura pelagica*	XP_009999706.1
71.	Cuckoo roller	*Leptosomus discolor*	XP_009954693.1
72.	Reptile bird	*Opisthocomus hoazin*	XP_009942673.1
73.	Killdeer	*Charadrius vociferus*	XP_009879047.1
74.	South African ostrich	*Struthio camelus australis*	XP_009688499.1
75.	Dalmatian pelican	*Pelecanus crispus*	XP_009487536.1
76.	Crested ibis	*Nipponia nippon*	XP_009467776.1
77.	Adelie penguin	*Pygoscelis adeliae*	XP_009328306.1
78.	Emperor penguin	*Aptenodytes forsteri*	XP_009277624.2
79.	Common canary	*Serinus canaria*	XP_018770832.1
80.	Carmine bee-eater	*Merops nubicus*	XP_008938515.1
81.	Polar beer	*Ursus maritimus*	XP_008689915.1
82.	Przewalski’s horse	*Equus przewalskii*	XP_008517941.1
83.	Anna’s hummingbird	*Calypte anna*	XP_008501640.1
84.	Green monkey	*Chlorocebus sabaeus*	XP_008012957.1
85.	Elephant shark	*Callorhinchus milii*	XP_007883233.1
86.	Western European hedgehog	*Erinaceus europaeus*	XP_016041855.1
87.	Yangtze river dolphin	*Lipotes vexillifer*	XP_007468003.1

Supplementary Table 4. Accession numbers of δ1 protocadherin protein sequences used for studying conservation of residues across different family members.

S/N	Name	Accession Number
1.	*hs* PCDH1	NP_115796.2
2.	*mm* PCDH1	NP_083633.2
3.	*hs* PCDH7	NP_001166994.1
4.	*mm* PCDH7	NP_001116230.1
5.	*hs PCDH9*	NP_982354.1
6.	*mm* PCDH9	NP_001074846.1
7.	*hs* PCDH11X	NP_116750.1
8.	*mm* PCDH11X	NP_001258738.1
