# Supplementary material for: Identification of an adhesive interface for the non-clustered δ1 protocadherin-1 involved in respiratory diseases
Source: Commun Biol. 2019 Sep 30;2:354. doi: 10.1038/s42003-019-0586-0 (PMC6769022; doi:10.1038/s42003-019-0586-0)
Supplement: Supplementary file 2 — Description of Additional Supplementary Files [file 42003_2019_586_MOESM2_ESM.docx]

**Supplementary Data 1**

**Bead Aggregation Assays Source File:** This Excel file contains source data for bead aggregation assays shown in Figures 1i, 5h, and Supplementary Figure 1h. The mean of means, standard deviation, and standard errors for the constructs are listed in the excel file.

**Supplementary Data 2**

**Analytical Ultracentrifugation Source File:** This Excel file contains source data for analytical ultracentrifugation experiments shown in Figures 2c, 2d, 5c, 5f, and Supplementary Figure 6f. Sedimentation coefficients, *c*(*s*), and normalized *c*(*s*) for each construct are listed.

**Supplementary Data 3**

**Differential Scanning Fluorimetry Source File:** This Excel file contains source data associated with the differential scanning fluorimetry experiments shown in Supplementary Figure 7. The temperatures, fluorescence, normalized fluorescence, average of the repeats, and standard deviations are listed for each construct.

**Supplementary Movie 1**

**Interface of a non-clustered δ1 protocadherin has more EC1-EC4 contacts:** Movie showing the antiparallel homophilic PCDH1 EC1-4 interface (δ1 protocadherin). Protomers are shown in scarlet and mauve molecular surface representations with bound calcium ions are shown as green spheres. The EC1-EC4 interface area is larger than the EC2-EC3 interface area.

**Supplementary Movie 2**

**Interface of a non-clustered δ2 protocadherin has more EC2-EC3 contacts:** Movie showing the antiparallel homophilic PCDH19 EC1-4 interface (δ2 protocadherin). Protomers are shown in light blue and cyan molecular surface representations with bound calcium ions shown as green spheres. The EC2-EC3 interface area is larger than the EC1-EC4 interface area.
