# Supplementary material for: Identification of an adhesive interface for the non-clustered δ1 protocadherin-1 involved in respiratory diseases
Source: Commun Biol. 2019 Sep 30;2:354. doi: 10.1038/s42003-019-0586-0 (PMC6769022; doi:10.1038/s42003-019-0586-0)
Supplement: Supplementary file 8 — Reporting Summary [file 42003_2019_586_MOESM8_ESM.pdf]

nature research

Corresponding author(s): Marcos Sotomayor

Last updated by author(s): Jul 24, 2019

Reporting Summary

Nature Research wishes to improve the reproducibility of the work that we publish. This form provides structure for consistency and transparency in reporting. For further information on Nature Research policies, see Authors & Referees and the Editorial Policy Checklist .

Statistics

For all statistical analyses, confirm that the following items are present in the figure legend, table legend, main text, or Methods section.

n/a	Confirmed	
☐	☒	The exact sample size (*n*) for each experimental group/condition, given as a discrete number and unit of measurement
☐	☒	A statement on whether measurements were taken from distinct samples or whether the same sample was measured repeatedly
☒	☐	The statistical test(s) used AND whether they are one- or two-sided<br>*Only common tests should be described solely by name; describe more complex techniques in the Methods section.*
☒	☐	A description of all covariates tested
☒	☐	A description of any assumptions or corrections, such as tests of normality and adjustment for multiple comparisons
☒	☐	A full description of the statistical parameters including central tendency (e.g. means) or other basic estimates (e.g. regression coefficient) AND variation (e.g. standard deviation) or associated estimates of uncertainty (e.g. confidence intervals)
☒	☐	For null hypothesis testing, the test statistic (e.g. *F*, *t*, *r*) with confidence intervals, effect sizes, degrees of freedom and *P* value noted<br>*Give P values as exact values whenever suitable.*
☒	☐	For Bayesian analysis, information on the choice of priors and Markov chain Monte Carlo settings
☒	☐	For hierarchical and complex designs, identification of the appropriate level for tests and full reporting of outcomes
☒	☐	Estimates of effect sizes (e.g. Cohen's *d*, Pearson's *r*), indicating how they were calculated

Our web collection on statistics for biologists contains articles on many of the points above.

Software and code

Policy information about availability of computer code

Data collection	X-ray diffraction data were collected remotely at the Argonne National Lab, beamline 24-ID-E using NE-CAT software. Data were indexed, integrated and scaled using HKL2000. Bead aggregation assay images were collected using a Nikon Eclipse Ti microscope with associated NIS-Elements BR 4.40 software. Differential scanning calorimetry experiments data were collected using an CFXConnect Rt-PCR machine with associated Bio-Rad CFX Manager 3.1 software. Analytical ultracentrifugation data were collected using a Proteome Lab XL-I ultracentrifuge with associated software Proteome Lab XL-I 6.0.
Data analysis	Geneious 11.1.4- For sequence analysis<br>ImageJ- For bead aggregation assay data analysis<br>Sedfit 1501b- For analytical ultracentrifugation data analysis<br>HKL2000- For indexing, integrating and scaling datasets.<br>CCP4i 7.0 (Scalepack2mtz, Matthews_coef, Phaser MR, Refmac5)- For structure solution and refinement<br>Coot 0.8.9.1 EL- For real space refinement of structures<br>VMD 1.9.3- For generating molecular images<br>OriginPro 8- For generating graphs<br>PyMOL 2.2.0- For generating electron density figures<br>ClustalX 2.1-For sequence alignments<br>Clustal Omega- For sequence alignments<br>SIAS- For generating similarity and identity matrices between different proteins sequences<br>Consurf- For analyzing conservation of residues across different protein sequences<br>NetNGlyc 1.0, NetOGlyc 4.0, NetCGlyc 1.0- For analyzing glycosylation<br>PISA- For prediction of interfaces in structures<br><br>All software packages listed above are freely or commercially available and have been described in associated publications cited in the manuscript.

For manuscripts utilizing custom algorithms or software that are central to the research but not yet described in published literature, software must be made available to editors/reviewers. We strongly encourage code deposition in a community repository (e.g. GitHub). See the Nature Research guidelines for submitting code & software for further information.

Data

Policy information about availability of data

All manuscripts must include a data availability statement. This statement should provide the following information, where applicable:

- Accession codes, unique identifiers, or web links for publicly available datasets
- A list of figures that have associated raw data
- A description of any restrictions on data availability

Coordinates for hs PCDH1 EC1-4bc, hs PCDH1 EC1-4mc, and hs PCDH1 EC3-4bc have been deposited in the Protein Data Bank with entry codes 6BX7, 6MGA an 6PIM respectively. There is no restriction on data availability.

Field-specific reporting

Please select the one below that is the best fit for your research. If you are not sure, read the appropriate sections before making your selection.

☒ Life sciences ☐ Behavioural & social sciences ☐ Ecological, evolutionary & environmental sciences

For a reference copy of the document with all sections, see nature.com/documents/nr-reporting-summary-flat.pdf

Life sciences study design

All studies must disclose on these points even when the disclosure is negative.

Sample size	Sample size was chosen as per previous work (Cooper et al., eLife 2016, Biswas et al., Journal of Cell Biology 2010).
Data exclusions	For the bead aggregation assays, the repeats for which there was no band for protein expression in western blots were excluded from analysis.
Replication	To verify the reproducibility of the experimental findings, bead aggregation assays and differential scanning fluorimetry experiments were repeated at least two times and analytical ultacentrifugation experiments were repeated twice. Same results were obtained each time.
Randomization	Not applicable
Blinding	Not applicable

Reporting for specific materials, systems and methods

We require information from authors about some types of materials, experimental systems and methods used in many studies. Here, indicate whether each material, system or method listed is relevant to your study. If you are not sure if a list item applies to your research, read the appropriate section before selecting a response.

Materials & experimental systems

n/a	Involved in the study	
☐	☒	Antibodies
☐	☒	Eukaryotic cell lines
☒	☐	Palaeontology
☒	☐	Animals and other organisms
☒	☐	Human research participants
☒	☐	Clinical data

Methods

n/a	Involved in the study	
☒	☐	ChIP-seq
☒	☐	Flow cytometry
☒	☐	MRI-based neuroimaging

Antibodies

Antibodies used	Rhodamine(TRITC)-conjugated AffiniPure Goat Anti-Human IgG(H+L)- Jackson Immunoresearch, Catalog- 109-025-003, Lot-117103<br>Mouse anti-goat IgG-HRP, Santa Cruz Biotechnology, Catalog-sc-2354, Lot-A2017
Validation	Both antibodies were used for western blots only as used in Cooper et al., eLIFE 2016.

Eukaryotic cell lines

Policy information about cell lines

Cell line source(s)	HEK 293T (ATCC® CRL-1573™)<br>Expi293 (ThermoFischer Scientific A14527)
Authentication	Cell lines were not authenticated as these were used for protein production only.
Mycoplasma contamination	Cell lines were not tested for mycoplasma contamination as these were used for protein production only.
Commonly misidentified lines<br>(See ICLAC register)	none
